# Building consensus on prescribing self‐directed occupational therapy activities: A Delphi study

**DOI:** 10.1111/1440-1630.70029

**Published:** 2025-06-08

**Authors:** Anna Joy, Alicia Devlin, Natasha A. Lannin, Libby Callaway, Sara L. Whittaker, Natasha K. Brusco

**Affiliations:** ^1^ Occupational Therapy Department Monash University Frankston Victoria Australia; ^2^ Rehabilitation, Ageing and Independent Living (RAIL) Research Centre Frankston Victoria Australia; ^3^ School of Translational Medicine Monash University Melbourne Victoria Australia

**Keywords:** implementation, occupational therapy, rehabilitation, self‐management

## Abstract

**Background:**

Occupational therapy practice focuses on occupation‐based interventions, considering the interaction between the person, environment, and task. In Australia, combining supervised therapy with self‐directed practice is feasible, even for health‐care consumers with cognitive impairments. This study aims to explore the self‐practice programs developed by occupational therapists and delivered in inpatient rehabilitation and develop core recommendations for such interventions.

**Methods:**

Ethical approval for this Delphi consensus study was granted by Monash University Human Research Ethics Committee. The study involved an online survey and two focus groups with Australian occupational therapists. Participants were recruited via snowball sampling and required to meet specific experience criteria. Content analysis was used to analyse data, and consensus was reached on core recommendations for prescribing self‐practice in inpatient rehabilitation.

**Consumer and community involvement:**

Occupational therapists who prescribe self‐practice to health‐care consumers were the community of interest and directly involved in the Delphi consensus process to inform study findings. Consumers using self‐practice activities were not included in the study.

**Results:**

In Round 1 of the Delphi process, 21 occupational therapists participated in an anonymous online survey about prescribing self‐practice in inpatient rehabilitation. The survey identified key recommendations across various categories. Round 2 focus groups further refined these recommendations, and Round 3 achieved consensus, incorporating additional feedback and suggestions for implementing self‐practice programs. It found variability in implementation, with neurological and general rehabilitation health‐care consumers most likely to receive self‐practice tasks.

**Conclusion:**

This Australian study explored how occupational therapists prescribe self‐practice during inpatient rehabilitation. The study emphasised the importance of clinical reasoning and environmental factors, offering recommendations to guide goal‐focused, client‐centred self‐practice interventions for better health‐care consumer outcomes.

**PLAIN LANGUAGE SUMMARY:**

We looked at occupational therapists in Australia. We wanted to know how therapists gave self‐practice activities to people. Our focus was on people in recovery hospitals. People do self‐practice activities without a therapist. People do these activities outside therapy sessions. This study used a survey and focus groups. Skilled therapists agreed on ideas for self‐practice. The results showed differences in self‐practice methods. Self‐practice is common in stroke and general recovery. The study gave self‐practice tips for therapists. It included what, how, and why they prescribe these activities. Therapists should think about each person's goals. They should understand what helps self‐practice. We know what therapists are doing. We support therapists to include self‐practice programs in regular care.

**Key Points for Occupational Therapy**

- Self‐directed practice prescribed by occupational therapists is empowering and motivating for health‐care consumers and can be part of usual care during inpatient rehabilitation.
- Guidance on how and what to prescribe as self‐practice is helpful to inform occupational therapy practice.
- Delphi consensus work indicated that occupational therapists take contextual factors into account when prescribing self‐directed practice.

## 1 INTRODUCTION

The nature of occupational therapy practice is such that an occupation‐centred perspective is core to clinical reasoning (Fisher, 2014; Fisher & Griswold, 2019). This lens adopts a client‐centred framework for assessment, goal setting, and intervention planning, encompassing the ability to analyse occupational performance by evaluating the interaction of the person, their environment and occupations they undertake (Fisher & Griswold, 2019; Townsend & Polatajko, 2007).

Occupational therapists assess and identify factors influencing occupational performance and recommend relevant occupation‐based interventions centred around the client's goals (Fisher & Griswold, 2019; Wall et al., 2023). Occupation‐based interventions may include whole task practice or part task practice (Fisher, 2014; Gray, 1998). Part practice involves intervention approaches that address components of a functional or occupation‐based task (i.e., breaking down the task into smaller activities) (Skubik‐Peplaski et al., 2017).

A range of treatment approaches inclusive of both supervised treatment and health‐care consumer self‐management via independent practice of tasks are feasible, including for people with cognitive impairment (Brusco et al., 2019, 2022, 2023, 2024). These treatment approaches cut across the World Health Organization (WHO) International Classification of Functioning, Disability, and Health (ICF) domains of body functions and structures (e.g., via upper extremity strength based exercises); activities and participation (e.g., via functional mobility or occupation‐based training); and environmental interventions (e.g., practising use of assistive products or home modifications) (World Health Organization, 2001). Although recent research has highlighted that occupation‐based interventions should be considered to improve occupational performance and participation outcomes within inpatient settings, the types of self‐practice tasks prescribed by occupational therapists is less evident in the literature (Wall et al., 2023).

The importance of participation in goal‐directed, meaningful activities prescribed by occupational therapists during inpatient rehabilitation is well recognised (Brusco et al., 2022, 2024; Wall et al., 2023). One of the challenges of inpatient rehabilitation is providing opportunity to practise therapy activities outside of structured sessions (Adey‐Wakeling et al., 2021; Brusco et al., 2019). Self‐directed practice of therapy programs is recommended for health‐care consumers during inpatient rehabilitation, with the aim to support self‐practice of therapy tasks outside formal therapy sessions (Brusco et al., 2019, 2023, 2024). Health‐care consumer participation in self‐directed therapy activity programs prescribed by occupational therapists and physiotherapists, such as the Australian‐based ‘My Therapy’ program, has been found to increase therapy participation by 26 minutes per day (Brusco et al., 2024; Whittaker et al., 2024). As part of the ‘My Therapy’ intervention, health‐care consumers complete a subset of therapeutic activities without supervision from the treating therapist, through a structured and documented program of self‐managed therapy practice aiming to educate, motivate, and empower health‐care consumers to undertake this practice independently and safely (Brusco et al., 2021). This self‐management approach is an encouraging step towards identifying additional strategies to provide health‐care consumers with the opportunity to reach the intensity of therapy that is recommended in current rehabilitation guidelines, alongside traditional supervised therapy practice as part of usual care (Australasian Faculty of Rehabilitation Medicine, 2019; Stroke Foundation, 2023). Adding to the findings from the My Therapy evaluation, other studies have also found self‐directed therapy programs across both general and stroke rehabilitation settings can result in additional functional improvements; however, improved reporting of intervention design and the use of robust measures of occupational performance have been encouraged (Harris, Eng, et al., 2009; Leung et al., 2018; Wall et al., 2023).

The growing body of evidence supporting the benefits of self‐practice programs in rehabilitation highlights the need for health professionals (including occupational therapists) to feel equipped and confident to provide such programs to complement existing models of delivery of therapeutic intervention (Brusco et al., 2022; Chin et al., 2022). Occupational therapy forms an important part of multidisciplinary health care within inpatient settings. However, self‐directed therapy activities are not currently part of routine occupational therapy care during inpatient rehabilitation (Brusco et al., 2023; Wall et al., 2023; Whittaker et al., 2024). Evidence exists to support the positive impact of occupation‐based interventions on a health‐care consumer's performance and participation outcomes (Murray et al., 2021). However, changes within the occupational therapy practice context have been identified as necessary, particularly focusing on intervention protocols and occupation‐enabled practice (Murray et al., 2021; Wall et al., 2023).

Understanding how and what occupational therapists prescribe as self‐practice within Australian rehabilitation settings is not well understood. This study therefore sought to explore expert opinions of experienced occupational therapists working in inpatient rehabilitation settings. The research had two aims: (1) to explore the self‐practice programs developed by occupational therapists and delivered in inpatient rehabilitation, including what is prescribed, why, and for which patient cohorts and (2) to develop a core set of recommendations to guide occupational therapy prescription of health‐care consumer self‐practice between supervised therapy sessions during inpatient rehabilitation.

## 2 METHODS

### 2.1 Human research ethics committee approval

Ethical approval for this study was granted by Monash University Human Research Ethics Committee (HREC), prior to the research commencing (project ID: 30269). The research consisted of participation in an online survey and two online focus groups, details of which are provided below. Study participants provided implied consent by commencing the online survey. Focus group participants provided signed consent for participation, using the HREC approved project explanatory statement and consent form.

### 2.2 Design

A Delphi consensus methodological design (O'Hara et al., 2000) was used to allow a consensus to be gained within a fixed timeframe, across a field of experienced occupational therapists working within Australian rehabilitation settings.

The three stages of the Delphi study included an online survey and two focus groups, used a collaborative consensus building process, and occurred in a sequential manner as follows:

1. Round 1: An online anonymous survey was used in the first round of the study. The survey design aimed to investigate and determine if occupational therapists currently prescribe self‐practice during inpatient rehabilitation as part of routine care. If they do, seek information on what is prescribed and why and for which inpatient cohorts. Based on available literature, a series of recommendations were presented for occupational therapists to rank their level of agreement. See Appendix A.
2. Round 2: An online focus group was hosted with participants who self‐nominated to participate in Round 2 at the end of the Round 1 survey. The focus group was used to discuss the value of self‐practice during inpatient rehabilitation, and what recommendations should guide occupational therapists when prescribing self‐directed therapy activities. The focus group allowed ideas to be further discussed, explored and evaluated (Allen et al., 2004).
3. Round 3: Researchers collated the responses from Round 2 for presentation at the (final) Round 3 focus group. The Round 3 focus group was to gain consensus on a final core set of recommendations (Diamond et al., 2014). See Table 2.

Occupational therapy recommendations for self‐practice were therefore developed, refined, prioritised, and agreed upon by study participants through these three rounds of data collection. Study results were reported in line with the Recommendations for the Conducting and Reporting of Delphi Studies (CREDES) (Jünger et al., 2017).

### 2.3 Participants

It has previously been reported that Delphi panel members should be highly specialised in the area of knowledge (Hsu & Sandford, 2007). However, it has also been recommended that there is value in panel members being heterogenous, rather than homogenous (Niederberger & Spranger, 2020; Powell, 2003). For this reason, a range of study inclusion criteria were specified, with participants needing to meet a minimum of at least two of these. All participants were included if they met the participant characteristics. Occupational therapists were therefore eligible to participate in this study if they were registered with the Australian Health Practitioner Regulation Agency (AHPRA) and met at least two of the following criteria specific to the expertise sought:

- A minimum of 3 years prescribing self‐practice exercises to health‐care consumers within inpatient rehabilitation settings;
- A minimum of 7 years of clinical experience;
- Employed in a senior clinician role working in an inpatient rehabilitation setting (as usually indicated by grade three/grade four, level four/level five, health practitioner four [HP4]/health practitioner five (HP5) depending upon the jurisdiction in Australia);
- Held a post‐graduate qualification in occupational therapy; and/or
- Provided clinical supervision to junior allied health professionals in a rehabilitation setting.

There is no consensus in the literature regarding the minimum number of participants for a Delphi study panel (Hasson et al., 2000). Some Delphi studies have included 12 or fewer panel members (Malone et al., 2005; Strasser et al., 2005) whereas other Delphi studies have included more than 30 panel members (Kelly & Porock, 2005; Meadows et al., 2005; Westby et al., 2014). The expert panel for this study aimed to include a minimum of 20 occupational therapists.

### 2.4 Participant recruitment

A purposive sampling method was initially used in this study. Occupational therapists who had their contact details within the public domain, and it could be determined by the research group that they meet the study inclusion criteria, were invited via email to participate. If the person did not wish to consider participation, they could disregard the email. If they wanted to hear more or consider consent to participation, they were invited to contact a nominated researcher via a return email, and they were then provided with the HREC‐approved project explanatory statement. Additional people were contacted through public domain occupational therapy practitioner directories and via public domain contact details of inpatient rehabilitation occupational therapy departments across Australia. A snowball sampling approach was then used for subsequent participant recruitment. A snowball approach is where participants who agreed to participate in the study are asked whether they can recommend other people who would also meet the inclusion criteria and may be contacted to explain the study and consider consenting to participation (Portney & Watkins, 2015). As snowball sampling may under‐represent smaller population groups, such as rural or sole practitioners (and therefore may produce biased samples), social media advertisements were also shared via the researchers' social media accounts with the aim to recruit eligible participants to complete the Round 1 survey and consider nominating for Rounds 2 and 3 focus group activities.

### 2.5 Data collection

The Round 1 anonymous online survey data were collected and managed using Research Electronic Data Capture (REDCap) electronic data capture tools hosted and managed by Helix (Monash University) (Harris et al., 2019; Harris, Taylor, et al., 2009). See Appendix A. There was the option for each participant to complete two additional survey questions asking whether they wanted to receive a copy of the study results via email and whether they were interested in being contacted to participate in a future focus group (Rounds 2 and 3 consensus work). Both of these questions launched in new REDCap surveys, as the questions required them to then enter their name and contact details, and thus identify themselves to the research group.

A subset of survey participants self‐nominated to participate in two (Rounds 2 and 3) online focus groups. The focus groups were structured to build on findings from the survey data, by exploring perceptions regarding the value of self‐practice during inpatient rehabilitation; consider recommendations that should guide occupational therapists when prescribing self‐directed therapy activities; and gain consensus on a final core set of recommendations. See Appendix B for a copy of the focus group question guide.

### 2.6 Data analyses

The survey data were analysed using an inductive process of content analysis (Portney & Watkins, 2015) with the survey findings then presented at the first focus group. Researchers (A. J. and A. D.) compiled findings from the Round 1 survey to establish a drafted core set of recommendations for testing in Round 2 consensus work. Each of the individual recommendations required approval from 75% (or more) of the participants in the Round 1 survey to progress to the drafted core set of recommendations used in Round 2. As such, the threshold for the Delphi process was determined when consensus had been reached by 75% of occupational therapists participating in the study, who agreed on the inclusion of a specific recommendation. Likewise, if a recommendation was not approved by 75% (or more) of the participants, it did not progress to the final draft core set of recommendations.

### 2.7 Positionality statement

All authors are experienced Australian occupational therapy or physiotherapy clinicians and researchers and have worked in the public health setting in leadership roles in rehabilitation. All authors have clinical experience with prescribing self‐practice occupational therapy activities in either public or private healthcare settings. This lens was considered at all stages during the project design, implementation, and analysis to minimise any bias introduced from the authors' experiences.

## 3 RESULTS

### 3.1 Round 1 Delphi process

Twenty‐one occupational therapists completed the anonymous online survey, as Round 1 of the Delphi process. Of these, 20 met the inclusion criteria for the study, with 1 occupational therapist's responses excluded from the dataset due to them not meeting the minimum of two of the inclusion criteria. An overall participation rate was unable to be determined for the study due to the nature of the snowballing recruitment strategy, with no finite population. Participants primarily worked in public health‐care settings (*n* = 18) and were located across five states within Australia (see Table 1). Specific to Delphi study rounds consensus work, respondents were able to provide multiple responses to each question (see Table 2).

**TABLE 1:** Demographic details of study participants contributing to the anonymous survey (Round 1 consensus work).

Demographic information: Survey respondents	*n* (%)
Years in profession	
1–5	3 (15)
6–10	6 (30)
11–15	3 (15)
16–20	2 (10)
21–25	5 (25)
26–30	1 (5)
Postgraduate qualification	
Yes	4 (20)
No	16 (80)
Provides supervision to others	
Yes	19 (95)
No	1 (5)
Grade^a^	
G1 Victoria	1 (5)
G2 Victoria	3 (15)
G3 Victoria	6 (30)
Level 3 NSW	1 (5)
Other NSW	1 (5)
HP4 Queensland	2 (10)
HP5 Queensland	1 (5)
Level 2 WA	2 (10)
Level 3 WA	1 (5)
Level 4 WA	1 (5)
Other ACT	1 (5)
State	
Victoria	10 (50)
NSW	2 (10)
Queensland	3 (15)
WA	4 (20)
SA	0 (0)
ACT	1 (5)
Tasmania	0 (0)
Clinical setting^b^	
Public health‐care setting	18 (90)
Private hospital	1 (5)
Private community practice	2 (10)
Age (years)	
20–29	4 (20)
30–39	8 (40)
40–49	5 (25)
50–59	1 (5)
Undisclosed	2 (10)

^a^ Grade categorised using: Allied Health Professionals (Victorian Public Sector) (Single Interest Employers) Enterprise Agreement 2021‐2016 (Victorian Government, 2021); NSW Health Service Health Professionals (State) Award 2023 (NSW Government, 2023); Health Practitioners and Dental Officers (Queensland Health) Certified Agreement (No. 4) 2022 (Queensland Health, 2022); WA Health System HSUWA PACTS Industrial Agreement 2022 (Western Australian Government, 2022).

^b^ More than one clinical setting may have been selected by participants.

**TABLE 2:** Delphi method consensus outcomes by round.

Area of guideline	Round 1 (*n* = 20 participants met inclusion criteria)	Round 2 (*n* = 3)	Round 3 (*n* = 3)	Total recommendations
Why	48 responses 10 proposed recommendations	10 recommendations discussed. −1 did not achieve consensus +1 recommendation added	10 recommendations gained consensus	10
Factors	70 responses 11 proposed recommendations	11 recommendations gained consensus +5 recommendations added	16 recommendations gained consensus	16
What	6 proposed recommendations	6 recommendations gained consensus +1 recommendation added from ‘areas of self‐practice’	6 recommendations gained consensus +2 added also gaining consensus	8
How	3 proposed recommendations	3 recommendations gained consensus +5 recommendations added	8 recommendations gained consensus	8
Areas of self‐practice prescribed	86 responses 12 areas of self‐practice	The 12 areas gained consensus. Combined into 1 recommendation. Moved under ‘what’ heading.	—	12

### 3.2 Self‐practice prescription as part of usual care (the current state)

The majority of respondents worked with a mixed caseload of clients (*n* = 17) and have prescribed self‐practice across a variety of clinical environments including trauma (*n* = 7), neurology (*n* = 14), orthopaedics (*n* = 13), ageing (*n* = 6), and general rehabilitation (*n* = 13). All respondents (*n* = 20) reported that they had prescribed exercise or activities for self‐practice during inpatient rehabilitation. Occupational therapists reported that they prescribed self‐practice to on average 55% (range 17%–100%) of health‐care consumers. Instructions for self‐practice activities were provided to inpatients using a range of methods including: handwritten instructions (*n* = 12); typed and printed out exercises (*n* = 16); verbal instruction (*n* = 17); prescription using online software programs (e.g., PhysioTherapy eXercises (Harvey et al., 2024) (*n* = 7); instructions sent via email (*n* = 4); and video self‐practice instructions (*n* = 1).

### 3.3 Therapy activities clinicians prescribe for self‐practice

After the iterative and independent process of content analysis undertaken by two researchers (A. J. and A. D.) separately, the 86 unique open‐text responses to ‘what do you prescribe’ were then reviewed, and a final 12 categories of self‐practice tasks were generated. These were self‐care tasks; meal preparation tasks; productivity tasks; pressure care strategies; computer‐generated exercise programs; accessing available facilities; upper limb exercises and activities; scar management; assistive technology practice; energy conservation strategies; functional mobility practice; and relaxation strategies.

### 3.4 Reasons clinicians prescribe self‐practice

Of the 48 individual open‐text responses provided in the survey to the question ‘why you prescribe self‐practice’, 23 unique reasons why clinicians prescribe self‐practice were generated using content analysis. Ten proposed recommendations were identified through an iterative process of content analysis independently applied by two researchers (A. J. and A. D.). These were to promote participation in everyday activities; increase the dosage of therapy; increase health‐care consumer confidence; support success and independence to carry into the community setting; target activities at the right level for health‐care consumers' current abilities; empower health‐care consumers to undertake activities outside of formal therapy sessions; offer opportunity to increase practice; build skills for self‐management on discharge and provide ownership; pass time or offer diversional therapy; and prevent complications.

### 3.5 Factors impacting prescription of self‐practice (barriers/facilitators)

Of the 70 open‐text responses to this question, 22 initial factors impacting the prescription of self‐practice were generated via independent and iterative content analysis, and then consensus work, by two researchers (A. J. and A. D.). These were compiled to 11 separate factors impacting prescription of self‐practice, which were the health‐care consumer's cognitive ability; their rehabilitation potential; whether an occupational therapist was required to be present for activity fidelity (i.e., to ensure the health‐care consumers completed activities in the way that they should be done); psychosocial factors for the health‐care consumer; their level of mobility; health‐care consumer goals; safety considerations; therapist perceptions of the health‐care consumer's time use; a health‐care consumer's ability to communicate with others and their primary spoken language; availability of supports, for example, family to assist with set‐up for self‐practice; and equipment available for self‐practice.

### 3.6 Agreement with literature informed self‐practice statements

Round 1 respondents generally agreed with literature‐informed self‐practice statements, with at least 85% of respondents agreeing with each literature statement in the survey. A full list of the literature statements and participant data are available in Table 3.

**TABLE 3:** Literature statement agreement results.

Literature statement and author	Number of agreements	Number of disagreements	Total
Biological factors must first be considered through an assessment process prior to exercise prescription (Palermo & Corrà, 2017).	18 (90)	2 (10)	20 (100)
Rehabilitation program goals must be considered when determining exercise prescription (Palermo & Corrà, 2017).	20 (100)	0 (0)	20 (100)
Exercise‐related hurdles must be determined of individuals when determining exercise prescription (Palermo & Corra, 2017).	17 (85)	3 (15)	20 (100)
Safety must be taken into consideration when determining exercise prescription (Palermo & Corrà, 2017).	19 (95)	1 (5)	20 (100)
Patients should be encouraged to continue with active task practice outside of scheduled therapy sessions. This could include strategies such as: self‐directed, independent practice; semi‐supervised and assisted practice involving family/friends, as appropriate (Stroke Foundation, 2023).	20 (100)	0 (0)	20 (100)
Demands on allied health staff time are higher for those that manage a complex caseload, e.g., people with dual diagnoses, challenging behaviour, bariatric patients, patients with infection control requirements, substance misuse and dementia (Australasian Faculty of Rehabilitation Medicine, 2019).	19 (95)	1 (5)	20 (100)
The provision of therapy on weekends is strongly recommended as it has been shown to increase functional independence, physical activity, quality of life and in some cases, reduce length of stay (Australasian Faculty of Rehabilitation Medicine, 2019).	18 (90)	2 (10)	20 (100)
Equipment/assistive technology: Based on the needs of the patient casemix, a service should appropriate equipment and assistive technology (Australasian Faculty of Rehabilitation Medicine, 2019).	20 (100)	0 (0)	20 (100)
There is a written rehabilitation plan for each patient based on an assessment. The plan is patient centred and states the person's needs and limitations as well as the goals. The plan is prepared by a multidisciplinary team with the active participation of the patient and family and includes provision for continuing care, review and discharge (Australasian Faculty of Rehabilitation Medicine, 2019).	19 (95)	1 (5)	20 (100)
Therapy targeted at other activities of daily living should be task‐specific, progressive and practised frequently, and incorporated by the entire health‐care team into routine activities … every day of the week rather than confined to lengthy sessions separated by long periods of inactivity. (Intercollegiate Stroke Working Party, 2016).	19 (95)	1 (5)	20 (100)
Increasing the dosage of therapy increases quality of life and functional independence outcomes in rehabilitation patients (Peiris et al., 2013).	18 (90)	2 (10)	20 (100)
The availability of financial resources to support additional allied health staffing to meet the suggested dosage of therapy is a common barrier (O'Brien et al., 2017).	20 (100)	0 (0)	20 (100)
Nursing staff practice with rehabilitation patients is impacted by nursing staff perceptions of nursing levels and competing demands on their time. This directly impacts the integration of rehabilitation strategies into nursing care (Clarke, 2014).	20 (100)	0 (0)	20 (100)
One mechanism of increasing the dosage of therapy for inpatients in a rehabilitation setting is to prescribe independent tasks and exercises that is both feasible and safe (Brusco et al., 2019).	20 (100)	0 (0)	20 (100)
Side effects of medication have shown to have an impact on an individual's ability to undertake rehabilitation (i.e., negative symptoms including nausea, drowsiness, interrupted electrolyte levels) (Clarke & Witham, 2017).	20 (100)	0 (0)	20 (100)
Therapist perceptions of their own role and being able to hand over the control of care to the patients is another important consideration in the effectiveness of self‐directed practice (Norris & Kilbride, 2014).	19 (95)	1 (5)	20 (100)

### 3.7 Additional considerations from the survey results

Other considerations respondents highlighted were categorised into what occupational therapists prescribe (*n* = 6) and how they prescribe (*n* = 3). What occupational therapists prescribe included self‐practice that is not a replacement for usual care; prescribing self‐directive activity as an addition to occupational therapy treatment delivered by the clinician; participation in activity is often informally encouraged by the occupational therapist but is not formalised; the importance of the therapist actively demonstrating the self‐practice task/s and ensuring that activities are being consistently completed in the correct way; clinicians will spend some therapy time setting up, monitoring, and revising the self‐directed activity program with the health‐care consumer during formal therapy sessions; and occupational therapists do not prescribe activities to pass time. How clinicians prescribed self‐practice activities included self‐directed therapy activity programs should be provided to the health‐care consumer in written or electronic format; a health‐care consumer completion record or tracking table should be included; and prescription of self‐directed therapy activity programs must be documented in the medical file.

### 3.8 Round 2 Delphi process

Participant numbers decreased from 20 to 3 between rounds, the reasons for which are unknown. Three of the 20 occupational therapists who completed the Round 1 survey entered their details to be contacted about and provided signed consent to participate in Rounds 2 and 3 focus groups. The three participants who participated in Round 2 were all employed in different public health settings and held roles as senior clinicians who provide supervision to other occupational therapists. The 42 proposed recommendations identified from Round 1 were divided into five categories. There were 6 recommendations on ‘what clinicians prescribe’; 10 recommendations on ‘why clinicians prescribe’; 3 recommendations on ‘how clinicians prescribe’; 11 recommendations on ‘factors impacting prescription’; and 12 recommendations on ‘areas of self‐practice prescribed’ discussed within the focus groups. In the Round 2 focus group, consensus was reached for 41 out of the 42 recommendations. Of the 10 recommendations on ‘why clinicians prescribe’, 9 recommendations gained consensus and were accepted by the group. One recommendation (‘to pass time or offer diversional therapy’) was not supported by 75% of participants and was therefore excluded from the list of recommendations. The literature‐informed self‐practice statements from the survey were further explored in the first focus group, particular to those where one or more participants disagreed with the statement. Through this dialogue, consensus was achieved for all literature statements. Further comments were invited at the end of the first focus group.

### 3.9 Additional recommendations generated

Iterative content analysis of these 13 comments was undertaken by two independent researchers separately, and then compared using a consensus process, resulted in six final recommendations grouped under headings of ‘why clinicians prescribe’ (*n* = 1) and ‘how to prescribe’ (*n* = 5). The additional single recommendation as to ‘why clinicians prescribe’ self‐practice was ‘as an addition to formal occupational therapy sessions to promote progress in between sessions’. The additional ‘how to prescribe’ recommendations included the following: The program should be reviewed by the therapist weekly; prescribed activities should be health‐care consumer‐centred and goal‐focused; therapists should provide a dosage recommendation for self‐practice; therapists should provide equipment or resources required by the health‐care consumer to complete the program; and a collaborative approach between disciplines to prescribe self‐practice programs should be adopted, where appropriate.

### 3.10 Round 3 Delphi process

The same three participants from Round 2 participated in Round 3. The revised recommendations were presented in Round 3 consensus work (i.e., the final focus group) after feedback from Round 2 had been incorporated. Further comments from study participants in Round 3 resulted in two additional comments for consideration relating to content of the recommendations. The two content areas gained consensus and were added to that ‘what’ section of the recommendations. These were that regular review of the prescribed program is critical and that the program needs to link to health‐care consumer goals. Consensus was achieved at each phase of the Delphi study (Table 2).

Additional comments were provided by clinicians during the two focus groups about what resources they felt would be helpful for occupational therapists to implement the recommendations. These comments included the following: clear definitions about the different types of self‐practice prescribed by occupational therapists, that is, by using the WHO ICF domains of bodily functions and structures, activities, and participation (World Health Organization, 2001); links to occupational therapy practice frameworks; clear rationale to justify the prescription of self‐practice; exploration of common barriers and potential strategies under factors impacting prescription in final recommendations; and templates for self‐practice prescription. Agreement was reached by Round 3 of consensus work for all recommendations. The final recommendations are included in Appendix C.

## 4 DISCUSSION

The research found that occupational therapists do not prescribe self‐practice tasks to all health‐care consumers. Its implementation drew on significant clinical reasoning, was offered in varied formats (including written, verbal, and video instructions), and was implemented dependent on the patient cohort.

Specific to inpatient cohorts, the current study found that neurological and general rehabilitation inpatients were most likely to have self‐practice prescribed by occupational therapists, compared with inpatients who were frail, older, and had a cognitive impairment. This may partly be explained by these neurological and general rehabilitation groups often receiving interventions focused on body functions and structures. Although improving specific impairments can lead to changes in occupational performance, occupation itself is neither the means to achieve independence nor the immediate goal (Wall et al., 2023). The current study highlighted that some occupation‐focused interventions were prescribed as part of the self‐practice program (Fisher, 2014). However, it was noted that while activity and participation were encouraged, they were not always consistently supported through formal prescription or documented in medical records. Regardless of whether the intervention was component‐ or occupation‐focused, occupational therapists in the current study highlighted the complex clinical reasoning required to make decisions about the suitability and format of self‐practice activity prescription across these interventions and how these related to the interaction of the person, their environment, and the task or occupations (Townsend & Polatajko, 2007).

A recent evidence review has highlighted occupation‐based interventions improve occupational performance and participation outcomes in the inpatient rehabilitation setting and are valued for their impact on both outcomes and experience (Wall et al., 2023). Although self‐practice activity prescription in the current study primarily was described in relation to the ICF domains of bodily functions and structures and the environment, study participants also identified the importance of linking component‐focused interventions to occupation‐based practice (Fisher & Griswold, 2019). Recommendations offered in the current study's consensus work provided key learnings that can assist inpatient occupational therapists to prescribe self‐practice exercises that are both goal‐focused and client‐centred, as well as encompassing all WHO ICF domains of body function and structures, activities, participation, personal factors, and factors relating to the environment (World Health Organization, 2001).

Specific to the environment, Murray et al.'s recent scoping review highlighted that both factors relating to the clinician's skill set—as well as environmental factors—influence occupational therapy practice (Murray et al., 2021). These authors outlined individual factors (such as a clinician's knowledge, understanding, or confidence) and environmental factors specific to the inpatient setting (including availability of space and resources, and the fast‐paced and discharge‐focused nature of hospital settings) influence occupational therapy interventions provided. Through consensus, the Delphi expert panel in the current study identified recommendations that can guide occupational therapists in the prescription of self‐practice programs during inpatient rehabilitation, as well as considering and minimising the impact of environmental factors, like available space and resources.

This study had some limitations. Although there was consensus from clinicians in this study around the existing literature, further research is required to investigate whether these statements were implemented into practice and factors identified as barriers to implementation. In the current study, the potential barriers were prompted by presenting the literature statements to participants for consideration about their acceptance of these evidence‐based statements pertaining to self‐practice and providing their reasoning around any disagreement. By addressing these factors within the recommendations, barriers to the prescription of self‐practice were identified and potential solutions addressed; however, responses may have been influenced by the presentation of these literature statements and the potential bias of disagreeing with the statement being viewed as a non‐evidence‐based clinician. Following, although recommendations for the number of participants in a Delphi study vary, the number of participants who completed the online survey who then consented to participate in the second and third rounds of this Delphi study (using focus group methods) was lower than anticipated, and this should be considered when interpreting and generalising the study results. However, the focus groups attendees did provide a high level of engagement across a range of areas of expertise and built on survey data provided in the first round of the study. Finally, it should also be noted that this study was undertaken with occupational therapists working in Australia, and thus, terminology used and scope of practice described is aligned with the Australian occupational therapy practice context. Future research that draws on international occupational therapy consensus would be of value. Additionally, perspectives from consumers who use self‐practice activities prescribed by occupational therapists would be beneficial for future research. Although the findings from a Delphi study design are considered to be a lower level of evidence compared with experimental studies, this study does offer guidance for clinicians working in rehabilitation until more robust research evidence emerges.

## 5 CONCLUSIONS

Although occupational therapists support the provision of self‐practice programs during inpatient rehabilitation and believe that these programs improve outcomes, there is variability in practice. Occupational therapists' clinical reasoning informing the recommendation and design of self‐practice programs is closely considered in relation to the interaction of the person, their environment, and the component tasks or occupations in focus for intervention. This Delphi study offers an important first step to understand, standardise, and support occupational therapists to embed patient self‐practice programs into usual care within inpatient rehabilitation settings, as well as practical guidance on considerations for implementation.

## AUTHOR CONTRIBUTIONS

Conceptualisation: Natasha K. Brusco. Data collection: Anna Joy and Alicia Devlin. All authors contributed to the design, methodology, data analysis/interpretation, writing, and review of the article (Anna Joy, Alicia Devlin, Natasha A. Lannin, Libby Callaway, Sara L. Whittaker and Natasha K. Brusco).

## CONFLICT OF INTEREST STATEMENT

The authors have no conflict to declare.

## Data Availability

The data that supports the findings of this study are available in the supplementary material of this article

## Appendix A Survey Questions

**Section 1: Demographic information**

1 Years of experience as an occupational therapist:
2 Currently AHPRA registered? YES/NO
3 Years of experience in prescribing rehabilitation exercises:
4 Post‐graduate qualifications (Graduate Certificate, Masters or PhD): YES/NO

Qualification level: Graduate Certificate/Masters/PhD

5 Provides supervision to junior clinicians: YES/NO
6 Current state of Australia you practise in:

Victoria/NSW/Queensland/Western Australia/South Australia/ACT/Tasmania

7 Grade level in current position:

Grade 3 (VIC)/Grade 4 (VIC)/Level 4 (NSW)/Level 5 (NSW)/HP4 (QLD)/HP5 (QLD)/Other _____

8 Clinical setting:
  Public hospital/private hospital/private practice
9 Age:

**Section 2: Current practice**

1. Have you ever prescribed occupational therapy exercises or activities for self‐practice between formal therapy sessions? YES/NO
2. What proportion of your patients would you prescribe exercises or activities for self‐practice between therapy sessions to? SCALE 0‐100.
3. Please select the populations of clients to whom you have prescribed exercises:
4. Trauma/Neurological/Orthopaedics/Ageing and Reablement/General Rehabilitation
5. Why do you prescribe exercises or activities for self‐practice between formal therapy sessions? List all reasons that apply.
6. What factors do you consider to help to choose whether or not to prescribe? Describe all factors that apply.
7. What do you prescribe? Provide examples.
8. How do you usually provide these instructions to patients? Choose all that apply.

Handwritten/Typed and printed out/Verbal instructions/Use online software and print out/Use an app/Email/Other _______________________

10 The World Health Organization framework, the International Classification of Function (ICF) encompasses body structure/function, activity and participation.
  In relation to your prescription for patients' self‐practice, what proportion of your prescription would you define as:
  - Activity (related to the execution of a task or activity)
  - Exercises (related to anatomical and physiological functions)
  - Participation (related to participation in everyday life task)
  (Percentage measures 0%–100%)

**Section 3: Current evidence statements**

1. Do you agree/disagree with the following literature statements? *If participant selects ‘no’ a comment box will appear ‘why not’?*

- Biological factors must first be considered through an assessment process prior to exercise prescription (Palermo & Corrà, 2017).
- Rehabilitation program goals must be considered when determining exercise prescription (Palermo & Corrà, 2017).
- Exercise‐related hurdles must be determined of individuals when determining exercise prescription (Palermo & Corrà, 2017).
- Safety must be taken into consideration when determining exercise prescription (Palermo & Corrà, 2017).
- Patients should be encouraged to continue with active task practice outside of scheduled therapy sessions. This could include strategies such as: self‐directed, independent practice; semi‐supervised and assisted practice involving family/friends, as appropriate (Stroke Foundation, 2023).
- Demands on allied health staff time are higher for those that manage a complex caseload, for example, people with dual diagnoses, challenging behaviour, bariatric patients, patients with infection control requirements, substance misuse, and dementia (Australasian Faculty of Rehabilitation Medicine, 2019).
- The provision of therapy on weekends is strongly recommended as it has been shown to increase functional independence, physical activity, quality of life and in some cases, reduce length of stay (Australasian Faculty of Rehabilitation Medicine, 2019).
- Equipment/assistive technology: based on the needs of the patient casemix, a service should provide appropriate equipment and assistive technology (Australasian Faculty of Rehabilitation Medicine, 2019).
- There is a written rehabilitation plan for each patient based on an assessment. The plan is patient centred and states the person's needs and limitations as well as the goals. The plan is prepared by a multidisciplinary team with the active participation of the patient and family and includes provision for continuing care, review and discharge (Australasian Faculty of Rehabilitation Medicine, 2019).
- Therapy targeted at other activities of daily living should be task‐specific, progressive and practised frequently, and incorporated by the entire health‐care team into routine activities … every day of the week rather than confined to lengthy sessions separated by long periods of inactivity (Intercollegiate Stroke Working Party, 2016).
- Increasing the dosage of therapy increases quality of life and functional independence outcomes in rehabilitation patients (Peiris et al., 2013).
- The availability of financial resources to support additional allied health staffing to meet the suggested dosage of therapy is a common barrier (O'Brien et al., 2017).
- Nursing staff practice with rehabilitation patients is impacted by nursing staff perceptions of nursing levels and competing demands on their time. This directly impacts the integration of rehabilitation strategies into nursing care (Clarke, 2014).
- One mechanism of increasing the dosage of therapy for inpatients in a rehabilitation setting is to prescribe independent tasks and exercises that is both feasible and safe (Brusco et al., 2019).
- Side effects of medication have shown to have an impact on an individual's ability to undertake rehabilitation (i.e., negative symptoms including nausea, drowsiness, and interrupted electrolyte levels) (Clarke & Witham, 2017).
- Therapist perceptions of their own role and being able to hand over the control of care to the patients is another important consideration in the effectiveness of self‐directed practice (Norris & Kilbride, 2014).

11 What recommendations would you make to guide occupational therapists prescribing exercise or activities for self‐practice between formal therapy sessions during inpatient rehabilitation?
12 Do you have any other comments about the prescription of activities or exercises for self‐practice between formal therapy sessions?

**Section 4: Follow up**

1. Thank you for participating in this survey. Do you wish to receive a copy of the results of this project? *If yes, you will be directed to a separate REDCap survey to record your contact information in order to maintain your anonymity with this survey response*.
2. 2.Do you want to be contacted to participate in a focus group to inform the next stage of this project (i.e. the development of a set of guidelines for occupational therapists working in rehabilitation)? *If yes, you will be directed to a separate REDCap survey to record your contact information in order to maintain your anonymity with this survey response*.

## Appendix B Focus group guide

**Table:** First focus group (Round 2 consensus work)

PART A—Introduction	
1. Consent	Confirmation that all consent forms have been received
2. Introduction	Introduction to one another Housekeeping, confidentiality of the focus groups Introduction to occupational therapy recommendations for exercises to be prescribed to patients for self‐practice between supervised therapy sessions during inpatient rehabilitation.
PART B—Findings from the survey	
3. Group discussion	Present and discuss general findings from the survey
PART C—Recommendations from the group for exercises to be prescribed to patients for self‐practice between supervised therapy sessions during inpatient rehabilitation	
4. Group discussion	Present and discuss recommendations from the survey Ask the group to present and discuss additional recommendations
PART D—Summary of the focus group and thank you for participation	
5. Focus group conclusion	Summary of the focus group Reminder of confidentiality Thank you to the participants for participation

**Table:** Second focus group (Round 3 consensus work)

PART A—Introduction	
1. Consent	Confirmation that all consent forms have been received
2. Introduction	Introduction to one another Housekeeping, confidentiality of the focus groups
PART B—Findings from the survey	
3. Group discussion	Present and discuss general findings from the results of focus group 1.
PART C—Recommendations from the group for exercises to be prescribed to patients for self‐practice between supervised therapy sessions during inpatient rehabilitation	
4. Group discussion	Present and discuss recommendations from the survey Ask the group to provide comments, present and discuss additional recommendations
PART D—Summary of the focus group and thank you for participation	

## Appendix C Clinician recommendations

**Table:** Recommendations

WHY do we prescribe self‐directed therapy to patients?
As an addition to formal therapy sessions to promote progress in between sessions To increase the dose of therapy (formal therapy and self‐practice combined) As an opportunity to increase practice To promote participation in everyday activities and routine on the ward To prevent complications such as falls or pressure injuries To empower patients to undertake activities outside of formal therapy sessions To target activities at the right level for the patients' current abilities To support success and independence for the patient to carry into the community setting To build patient skills to self‐manage their own program on discharge and provide ownership To increase patient confidence.
WHAT do we prescribe?
Self‐practice is not a replacement for 1:1 therapy, patients should still receive usual care Prescribing self‐directive activity is in addition to usual therapy Participation in activity is often informally encouraged by the occupational therapist, but often is not formalised or provided in written format to the patient The therapist will be actively involved in demonstrating and ensuring that the activities are being completed in the correct way (fidelity) Clinicians will spend some therapy time setting up, monitoring and revising the self‐directed activity program with the patient during formal therapy sessions The prescribed activities should be individualised to align with each patient's goals We do not prescribe activities to pass time (i.e., diversional therapy) It is important that therapists regularly review the prescribed activities
HOW do we prescribe self‐directed therapy to patients?
Self‐directed therapy activity programs should be provided to client in written or electronic format Patient completion record or tracking table should be included Program to be reviewed by therapists weekly (or as your service determines) Prescription of self‐directed therapy activity programs must be documented in the medical file Prescribed activities should be patient centred, goal focused and able to be completed independently and safely by patient or with the support person Therapists should provide a dosage recommendation but it will be up to the patient how often and when they complete their program Therapists provide any equipment or resources needed to complete the program Treating occupational therapist and other allied health disciplines are responsible for prescribing the program. A collaborative approach should be adopted by clinicians where appropriate
FACTORS impacting prescription of self‐practice (barriers/facilitators)
Cognition Communication impairment Fatigue Fidelity Goals achieved for discharge Level of mobility Medical status Patient isolated Patient motivation Patients from a non‐English speaking background Patient time use Rehabilitation potential Safety (falls risk) Available clinician time Vision impairment Available equipment/space/resources/environment
AREAS of self‐practice prescribed
Self‐care tasks Meal preparation tasks Productivity tasks Pressure care strategies Computer‐generated exercise programs Accessing available facilities Upper limb exercises and activities Scar management strategies Assistive technology practice Energy conservation strategies Functional mobility practice Relaxation strategies

## References

[aot70029-bib-0001] Adey‐Wakeling, Z. , Jolliffe, L. , O'Shannessy, E. , Hunter, P. , Morarty, J. , Cameron, I. D. , Liu, E. , & Lannin, N. A. (2021). Activity, participation, and goal awareness after acquired brain injury: A prospective observational study of inpatient rehabilitation. Annals of Rehabilitation Medicine, 45(6), 413–421. 10.5535/arm.21034 35000366 PMC8743846

[aot70029-bib-0002] Allen, J. , Dyas, J. , & Jones, M. (2004). Building consensus in health care: A guide to using the nominal group technique. British Journal of Community Nursing, 9(3), 110–114. 10.12968/bjcn.2004.9.3.12432 15028996

[aot70029-bib-0003] Australasian Faculty of Rehabilitation Medicine . (2019). Standards for the provision of inpatient adult rehabilitation medicine services in public and private hospitals. https://www.racp.edu.au/docs/default-source/advocacy-library/afrm-standards-for-the-provision-of-inpatient-adult-rehabilitation-medicine-services-in-public-and-private-hospitals.pdf?sfvrsn=4690171a_4 10.1016/j.jphys.2021.02.008 33744190

[aot70029-bib-0004] Brusco, N. K. , Ekegren, C. L. , Morris, M. E. , Hill, K. D. , Lee, A. L. , Somerville, L. , Lannin, N. A. , Abdelmotaleb, R. , Callaway, L. , Whittaker, S. L. , & Taylor, N. F. (2024). Outcomes of the My Therapy self‐management program in people admitted for rehabilitation: A stepped wedge cluster randomized clinical trial. Annals of Physical and Rehabilitation Medicine, 67(8), 101867. 10.1016/j.rehab.2024.101867 39173328

[aot70029-bib-0005] Brusco, N. K. , Ekegren, C. L. , Taylor, N. F. , Hill, K. D. , Lee, A. L. , Somerville, L. , Lannin, N. A. , Abdelmotaleb, R. , Callaway, L. , Whittaker, S. L. , & Morris, M. E. (2021). Self‐managed occupational therapy and physiotherapy for adults receiving inpatient rehabilitation (‘My Therapy’): Protocol for a stepped‐wedge cluster randomised trial. BMC Health Services Research, 21(1), 811. 10.1186/s12913-021-06442-9 34384427 PMC8361638

[aot70029-bib-0006] Brusco, N. K. , Kugler, H. , Dufler, F. , Lee, A. L. , Walpole, B. , Morris, M. E. , Hill, K. D. , Ekegren, C. L. , Whittaker, S. L. , & Taylor, N. F. (2022). Including exercise self‐management as part of inpatient rehabilitation is feasible, safe, and effective for patients with cognitive impairment. Journal of Rehabilitation Medicine, Clinical Communications, 5, 1000076. 10.2340/20030711-1000076 35154583 PMC8771766

[aot70029-bib-0007] Brusco, N. K. , Tilley, L. , Walpole, B. , Kugler, H. , Li, R. , Kennedy, E. , & Morris, M. E. (2019). Feasibility of increasing the dosage of inpatient occupational therapy and physiotherapy rehabilitation via independent tasks and exercises. Australian Occupational Therapy Journal, 66, 739–752. 10.1111/1440-1630.12614 31602693

[aot70029-bib-0008] Brusco, N. K. , Walpole, B. , Kugler, H. , Tilley, L. , Thwaites, C. , Devlin, A. , Dorward, E. , Dulfer, F. , Lee, A. L. , Morris, M. E. , Taylor, N. F. , Dawes, H. , Whittaker, S. L. , & Ekegren, C. L. (2023). Barriers and facilitators to implementing self‐directed therapy activities in inpatient rehabilitation settings. Australian Occupational Therapy Journal, 70(5), 617–626. 10.1111/1440-1630.12891 37291993

[aot70029-bib-0009] Chin, L. F. , Rosbergen, I. C. M. , Hayward, K. S. , & Brauer, S. G. (2022). A self‐directed upper limb program during early post‐stroke rehabilitation: A qualitative study of the perspective of nurses, therapists and stroke survivors. PLoS ONE, 17(2), e0263413. 10.1371/journal.pone.0263413 35120167 PMC8815971

[aot70029-bib-0010] Clarke, C. L. , & Witham, M. D. (2017). The effects of medication on activity and rehabilitation of older people—Opportunities and risks. Advances in Rehabilitation Science and Practice, 6, 1–7. 10.1177/11795572717711433

[aot70029-bib-0011] Clarke, D. (2014). Nursing practice in stroke rehabilitation: Systematic review and meta‐ethnography. Journal of Clinical Nursing, 23(9–10), 1201–1226. 10.1111/jocn.12334 24102924

[aot70029-bib-0012] Diamond, I. R. , Grant, R. C. , Feldman, B. M. , Ling, S. C. , Moore, A. M. , & Wales, P. W. (2014). Defining consensus: A systematic review recommends methodologic criteria for reporting of Delphi studies. Journal of Clinical Epidemiology, 67(4), 401–409. 10.1016/j.jclinepi.2013.12.002 24581294

[aot70029-bib-0013] Fisher, A. G. (2014). Occupation‐centred, occupation‐based, occupation‐focused: Same, same or different? Scandinavian Journal of Occupational Therapy, 21(sup1), 96–107. 10.3109/11038128.2014.952912 25116751

[aot70029-bib-0014] Fisher, A. G. , & Griswold, L. A. (2019). Performance skills: Implementing performance analyses to evaluate quality of occupational performance. In B. B. Schell , G. Gillen , M. Scaffa , & E. Cohn (Eds.), Willard and Spackman's occupational therapy (13th ed.) (pp. 335–350). Lippincott Williams & Wilkins.

[aot70029-bib-0015] Gray, J. M. (1998). Putting occupation into practice: Occupation as ends, occupation as means. American Journal of Occupational Therapy, 52(5), 354–364. 10.5014/ajot.52.5.354 9588260

[aot70029-bib-0016] Harris, J. E. , Eng, J. J. , Miller, W. C. , & Dawson, A. S. (2009). A self‐administered graded repetitive arm supplementary program (GRASP) improves arm function during inpatient stroke rehabilitation: A multi‐site randomized controlled trial. Stroke, 40(6), 2123–2128. 10.1161/STROKEAHA.108.544585 19359633

[aot70029-bib-0017] Harris, P. A. , Taylor, R. , Minor, B. L. , Elliott, V. , Fernandez, M. , O'Neal, L. , McLeod, L. , Delacqua, G. , Delacqua, F. , Kirby, J. , & Duda, S. N. (2019). REDCap consortium, the REDCap consortium: Building an international community of software partners. Journal of Biomedical Informatics, 95, 103208. 10.1016/j.jbi.2019.103208 31078660 PMC7254481

[aot70029-bib-0018] Harris, P. A. , Taylor, R. , Thielke, R. , Payne, J. , Gonzalez, N. , & Conde, J. G. (2009). Research electronic data capture (REDCap)—A metadata‐driven methodology and workflow process for providing translational research informatics support. Journal of Biomedical Informatics, 42(2), 377–381. 10.1016/j.jbi.2008.08.010 18929686 PMC2700030

[aot70029-bib-0019] Harvey, L. , Glinsky, J. , Messenger, P. , and PTX Collaboration . (2024) Physiotherapy exercises. Accessed November 2024. www.physiotherapyexercises.com

[aot70029-bib-0020] Hasson, F. , Keeney, S. , & McKenna, H. (2000). Research guidelines for the Delphi survey technique. Journal of Advanced Nursing, 32(4), 1008–1015. 10.1046/j.1365-2648.2000.t01-1-01567.x 11095242

[aot70029-bib-0021] Hsu, C. , & Sandford, B. (2007). The Delphi technique: Making sense of consensus. Practical Assessment, Research & Evaluation, 12(10), 10. 10.7275/pdz9-th90

[aot70029-bib-0022] Intercollegiate Stroke Working Party . (2016). National clinical guideline for stroke for the UK and Ireland www.strokeguideline.org

[aot70029-bib-0023] Jünger, S. , Payne, S. A. , Brine, J. , Radbruch, L. , & Brearley, S. G. (2017). Guidance on conducting and reporting DElphi studies (CREDES) in palliative care: Recommendations based on a methodological systematic review. Palliative Medicine, 31(8), 684–706. 10.1177/0269216317690685 28190381

[aot70029-bib-0024] Kelly, K. , & Porock, D. (2005). A survey of pediatric oncology nurses' perceptions of parent educational needs. Journal of Pediatric Oncology Nursing, 22(1), 58–66. 10.1177/1043454204272537 15574727

[aot70029-bib-0025] Leung, J. , Fereday, S. , Sticpewich, B. , & Hanna, J. (2018). Extra practice outside therapy sessions to maximize training opportunity during inpatient rehabilitation after traumatic brain injury. Brain Injury, 32(7), 915–925. 10.1080/02699052.2018.1469046 29718728

[aot70029-bib-0026] Malone, D. , Abarca, J. , Hansten, P. , Grizzle, A. , Armstrong, E. , Bergen, R. , Duncan‐Edgar, B. , Solomon, S. , & Lipton, R. (2005). Identification of serious drug‐drug interactions: Results of the partnership to prevent drug‐drug interactions. The American Journal of Geriatric Pharmacotherapy, 3(2), 65–76. 10.1331/154434504773062591 15098848

[aot70029-bib-0027] Meadows, A. , Maine, L. , Keyes, E. , Pearson, K. , & Finstuen, K. (2005). Pharmacy executive leadership issues and associated skills, knowledge, and abilities. Journal of the American Pharmacists Association, 45(1), 55–62. 10.1331/1544345052843183 15730118

[aot70029-bib-0028] Murray, A. , Di Tommaso, A. , Molineux, M. , Young, A. , & Power, P. (2021). Contemporary occupational therapy philosophy and practice in hospital settings. Scandinavian Journal of Occupational Therapy, 28(3), 213–224. 10.1080/11038128.2020.1750691 32356478

[aot70029-bib-0029] Niederberger, M. , & Spranger, J. (2020). Delphi technique in health sciences: A map. Frontiers in Public Health, 8, 457. 10.3389/fpubh.2020.00457 33072683 PMC7536299

[aot70029-bib-0030] Norris, M. , & Kilbride, C. (2014). From dictatorship to a reluctant democracy: Stroke therapists talking about self‐management. Disability and Rehabilitation, 36(1), 32–38. 10.3109/09638288.2013.776645 23594054 PMC3906249

[aot70029-bib-0031] NSW Government . 2023 NSW health service health professionals (state) award 2023 NSW Government

[aot70029-bib-0032] O'Brien, L. , Mitchell, D. , Skinner, E. H. , Haas, R. , Ghaly, M. , McDermott, F. , May, K. , & Haines, T. (2017). What makes weekend allied health services effective and cost‐effective (or not) in acute medical and surgical wards? Perceptions of medical, nursing, and allied health workers. BMC Health Services Research, 17(1), 345. 10.1186/s12913-017-2279-z 28494806 PMC5427575

[aot70029-bib-0033] O'Hara, L. , De Souza, L. H. , & Ide, L. (2000). A Delphi study of self‐care in a community population of people with multiple sclerosis. Clinical Rehabilitation, 14(1), 62–71. 10.1191/026921500666135189 10688346

[aot70029-bib-0034] Palermo, P. , & Corrà, U. (2017). Exercise prescriptions for training and rehabilitation in patients with heart and lung disease. Annals of the American Thoracic Society, 14(Supplement_1), S59–S66. 10.1513/AnnalsATS.201702-160FR 28719753

[aot70029-bib-0035] Peiris, C. L. , Shields, N. , Brusco, N. K. , Watts, J. J. , & Taylor, N. F. (2013). Additional Saturday rehabilitation improves functional independence and quality of life and reduces length of stay: A randomized controlled trial. BMC Medicine, 11(1), 198. 10.1186/1741-7015-11-198 24228854 PMC3844491

[aot70029-bib-0036] Portney, L. G. , & Watkins, M. P. (2015). Foundations of clinical research (3rd ed.). F.A. Davis Company.

[aot70029-bib-0037] Powell, C. (2003). The Delphi technique: Myths and realities. Journal of Advanced Nursing, 41(4), 376–382. 10.1046/j.1365-2648.2003.02537.x 12581103

[aot70029-bib-0038] Queensland Health . (2022). Health practitioners and dental officers (Queensland Health) certified agreement (No. 4) 2022. Queensland Government.

[aot70029-bib-0039] Skubik‐Peplaski, C. , Custer, M. , Powell, E. , Westgate, P. M. , & Sawaki, L. (2017). Comparing occupation‐based and repetitive task practice interventions for optimal stroke recovery: A pilot randomized trial. Physical & Occupational Therapy in Geriatrics, 35(3–4), 156–168. 10.1080/02703181.2017.1342734

[aot70029-bib-0040] Strasser, S. , London, L. , & Kortenbout, E. (2005). Developing a competence framework and evaluation tool for primary care nursing in South Africa. Education and Health, 18(2), 133–144. 10.1080/13576280500145615 16009609

[aot70029-bib-0041] Stroke Foundation . (2023). Clinical guidelines for stroke management. https://informme.org.au/guidelines/living-clinical-guidelines-for-stroke-management

[aot70029-bib-0042] Townsend, E. A. , & Polatajko, H. J. (2007). Enabling occupation II: Advancing an occupational therapy vision for health, well‐being, & justice through occupation. CAOT Publications ACE.

[aot70029-bib-0043] Victorian Government . (2021). Allied health professionals (Victorian public sector) (single interest employers) enterprise agreement 2021–2026. Victorian Government

[aot70029-bib-0044] Wall, G. , Isbel, S. , Gustafsson, L. , & Pearce, C. (2023). Occupation‐based interventions to improve occupational performance and participation in the hospital setting: A systematic review. Disability and Rehabilitation, 46(13), 2747–2768. 10.1080/09638288.2023.2236021 37524307

[aot70029-bib-0045] Westby, M. D. , Brittain, A. , & Backman, C. L. (2014). Expert consensus on best practices for post‐acute rehabilitation after total hip and knee arthroplasty: A Canada and United States delphi study. Arthritis Care and Research, 66(3), 411–423. 10.1002/acr.22164 24023047

[aot70029-bib-0046] Western Australian Government . (2022). WA health system HSUWA PACTS industrial agreement 2022. Western Australian Government

[aot70029-bib-0047] Whittaker, S. L. , Brusco, N. K. , Hill, K. D. , Ekegren, C. L. , & Taylor, N. F. (2024). A self‐management program increases the dosage of inpatient rehabilitation by 26 minutes per day: A process evaluation. Disability and Rehabilitation, 47(2), 425–434. 10.1080/09638288.2024.2339533 38627962

[aot70029-bib-0048] World Health Organization . (2001) ICF: International classification of functioning, disability and health World Health Organization Geneva World Health Organization

